# Age and SPARC Change the Extracellular Matrix Composition of the Left Ventricle

**DOI:** 10.1155/2014/810562

**Published:** 2014-03-24

**Authors:** Lisandra E. de Castro Brás, Hiroe Toba, Catalin F. Baicu, Michael R. Zile, Susan T. Weintraub, Merry L. Lindsey, Amy D. Bradshaw

**Affiliations:** ^1^Mississippi Center for Heart Research, University of Mississippi Medical Center (UMMC), Jackson, MS 39216, USA; ^2^San Antonio Cardiovascular Proteomics Center, University of Texas Health Science Center at San Antonio (UTHSCA), San Antonio, TX 78229, USA; ^3^Department of Clinical Pharmacology, Division of Pathological Sciences, Kyoto Pharmaceutical University, Kyoto, Japan; ^4^Gazes Cardiac Research Institute, Division of Cardiology, Department of Medicine, Medical University of South Carolina, Charleston, SC 29425, USA; ^5^Ralph H. Johnson Department of Veterans Affairs Medical Center, Charleston, SC 29401, USA; ^6^Department of Biochemistry, UTHSCSA, San Antonio, TX 78229, USA; ^7^Research Service, G.V. (Sonny) Montgomery Veterans Affairs Medical Center, Jackson, MS 39216, USA

## Abstract

Secreted protein acidic and rich in cysteine (SPARC), a collagen-binding matricellular protein, has been implicated in procollagen processing and deposition. The aim of this study was to investigate age- and SPARC-dependent changes in protein composition of the cardiac extracellular matrix (ECM). We studied 6 groups of mice (*n* = 4/group): young (4-5 months old), middle-aged (11-12 m.o.), and old (18–29 m.o.) C57BL/6J wild type (WT) and SPARC null. The left ventricle (LV) was decellularized to enrich for ECM proteins. Protein extracts were separated by SDS-PAGE, digested in-gel, and analyzed by HPLC-ESI-MS/MS. Relative quantification was performed by spectral counting, and changes in specific proteins were validated by immunoblotting. We identified 321 proteins, of which 44 proteins were extracellular proteins. Of these proteins, collagen III levels were lower in the old null mice compared to WT, suggestive of a role for SPARC in collagen deposition. Additionally, fibrillin showed a significant increase in the null middle-aged group, suggestive of increased microfibril deposition in the absence of SPARC. Collagen VI increased with age in both genotypes (>3-fold), while collagen IV showed increased age-associated levels only in the WT animals (4-fold, *P* < 0.05). These changes may explain the previously reported age-associated increases in LV stiffness. In summary, our data suggest SPARC is a possible therapeutic target for aging induced LV dysfunction.

## 1. Introduction 

Age is a prominent risk factor for increased cardiovascular morbidity and mortality, and the incidence of cardiovascular diseases such as hypertension and myocardial infarction (MI) are higher in individuals over 65 years of age [1]. Aging has been associated with significant structural changes in the left ventricle (LV) and data from several clinical trials show evidence that elderly patients have poorer outcomes after ischemic stress [2, 3]. The age-related decline in function of the cardiovascular system is associated with myocyte loss and a subsequent increase in the cardiac extracellular matrix (ECM) [4–6]. Evolving evidence suggests that cardiac senescence by itself affects myocardial structure and function that can affect how one responds to additional cardiac stressors. However, the frequent presence of comorbidities has hindered the identification of cardiac age-related therapeutic targets.

Myocardial ECM is composed of collagens, proteoglycans, glycoproteins, extracellular proteases, and ECM receptors [7, 8]. Among cardiac ECM components, fibrillar collagens provide myocardial structural support, mechanical stability, and morphology [9]. The collagenous matrix supports and aligns cardiomyocytes and vasculature and coordinates cell migration and proliferation [9, 10]. Experimental models have shown that excessive accumulation of fibrillar collagens in aged animals can lead to a significant decline in diastolic function [11, 12]. The major fibrillar collagens present in the myocardial ECM are collagen type I (approximately 85%, percentage is species dependent) and collagen type III (approximately 11%) [13]. These collagens display high tensile strength, which plays an important role in LV function [13]. Several experimental studies have clearly demonstrated a direct relation between increased collagen content and increased myocardial stiffness [14–16]. The factors responsible for increased collagen content with age are not well understood. However, cardiac aging is characterized by a loss of cardiomyocytes, and this may explain the increased collagen deposition in the LV walls [17]. Other factors that may be involved in age-associated fibrosis are inhibition of collagen degradation by changes in cardiac matrix metalloproteinases and respective tissue inhibitors of metalloproteinases and increased collagen fibril crosslinking and assembly [18].

Secreted protein acidic and rich in cysteine (SPARC), a matricellular protein, is known to regulate collagen fibril morphology and assembly [19]. SPARC has also been reported to regulate various cellular processes, including cell migration, proliferation, tissue morphogenesis, and tissue repair [20, 21]. In a mouse model, aging was associated with increased expression of SPARC and increased insoluble and fibrillar collagen content, which related to increased cardiac stiffness [2]. SPARC deletion blunted the aging-related effects. To further study the impact of this matricellular protein in cardiac ECM and aging, we developed an enrichment protocol coupled with a proteomic approach to analyze SPARC and age-dependent changes in the expression and accumulation of cardiac ECM proteins. This proteomics study used a mass spectrometry (MS) approach to identify low abundant ECM proteins in the cardiac matrix and immunoblotting to quantify protein changes that occur with age that are SPARC dependent. We tested the hypotheses that cardiac ECM protein content is influenced by progressive aging and that SPARC plays a key role in the structure and composition of the aged myocardium.

## 2. Materials and Methods

### 2.1. Animals and LV Collection

Mice colonies were maintained at the Medical University of South Carolina (MUSC) animal care facility. All procedures were performed in strict accordance with the Guide for the Care and Use of Laboratory Animals (National Research Council, The National Academies Press, Washington, DC, 2011) and were approved by the Institutional Animal Care and Use Committee at MUSC (Approval ID: ACORP 511). We used C57BL/6 wild type (WT) and SPARC-null mice to study age, sex, and genotype differences in the left ventricle (LV). Three age groups were studied: young (4-5 month old), middle-aged (11-12 month old), and old (18–29 month old), and both male and female mice were included in each group (*n* = 4/sex/age/genotype). The generation and phenotype of SPARC-null mice have been previously described by Norose and colleagues [22]. Animals were anesthetized with isoflurane and hearts excised. The hearts were washed in phosphate saline buffer (PBS) and the LV separated from the right ventricle. The LV was used for all further studies.

### 2.2. Tissue Decellularization

Whole LVs were decellularized as previously reported [23]. In summary, the tissue was incubated in decellularization buffer (1% sodium dodecyl sulfate in PBS) with 1x protease inhibitors cocktail (PI; cOmplete Mini tablets, Roche). Samples were left at room temperature in an orbital shaker until tissue was completely decellularized (three to four days). The decellularization buffer was decanted daily and replaced with fresh decellularization buffer. When tissue looked translucent, samples were considered decellularized. Tissue was washed three times in distilled water with 1x PI for 5 min and then left in fresh 1x PI/water overnight to remove all remnants of the decellularization buffer. The decellularized LVs were homogenized (Power Gen 1000, Fisher Scientific) in Protein Extraction Reagent Type 4 (7.0 M urea, 2.0 M thiourea, 40 mM Trizma base, and 1.0% C7BzO, pH 10.4) and 1x PI. Protein quantification was performed using a Coomassie Brilliant Blue G-250-based assay (Quick Start Bradford Protein Assay, Bio-Rad). All samples were stored at −80°C until use.

### 2.3. Mass Spectrometry

Proteins (10 *μ*g, *n* = 4 per age group/genotype) were separated by 1D SDS-PAGE in a 4–12% Bis-Tris gel and stained with EZBlue (Sigma Aldrich), which is a Coomassie Brilliant Blue-based dye compatible with MS analysis. The gel lane for each sample was divided into three slices, which contained the visually detectable proteins. Each slice was individually destained and dehydrated, and the proteins digested* in situ* with trypsin (Promega). The digests were analyzed by capillary HPLC-electrospray ionization tandem mass spectrometry (HPLC-ESI-MS/MS) on a Thermo Fisher LTQ Orbitrap Velos mass spectrometer fitted with a New Objective Digital PicoView 550 NanoESI source. Online HPLC separation of the digests was accomplished with an Eksigent/AB Sciex NanoLC-Ultra 2D HPLC system: column, PicoFrit (New Objective; 75 *μ*m i.d.) packed to 15 cm with C18 adsorbent (Vydac; 218 MS 5 *μ*m, 300 Å). Precursor ions were acquired in the Orbitrap in profile mode at 60,000 resolution (*m*/*z* 400); data-dependent collision-induced dissociation (CID) spectra of the six most intense ions in the precursor scan above a set threshold were acquired sequentially in the linear trap at the same time as the precursor ion scan. Mascot (version 2.3.02; Matrix Science) was used to search the mass spectra against a combination of the mouse subset of the NCBInr database (Mus. (145,083 sequences)) and a database of common contaminants (179 sequences). Methionine oxidation was considered as a variable modification; trypsin was specified as the proteolytic enzyme, with one missed cleavage allowed. The Mascot data files were combined in Scaffold (Proteome Software; version 3) for a subset search of the mass spectra using X! Tandem, cross correlation of the X! Tandem and Mascot results, and determination of probabilities of peptide assignments and protein inferences. The thresholds for acceptance of peptide and protein assignments in Scaffold were 95% and 99%, respectively, and minimum of one unique peptide.

### 2.4. Immunoblots

An aliquot of each sample (10 *μ*g protein) was loaded onto a 4–12% Bis-Tris gel and separated by 1D SDS-PAGE electrophoresis. Proteins were transferred to a nitrocellulose membrane, which was treated with the MemCode Reversible Protein Stain Kit (Pierce, Thermo Scientific) to check for efficiency of protein transfer and for use as a loading control. Destained membranes were blocked for 1 h at room temperature with 5% nonfat milk (Bio-Rad) and hybridized overnight at 4°C with primary antibodies against the following: collagen types I, III, IV, and VI (Cedarlane CL50141AP-1, CL50341AP-1, CL50441AP-1, and Abcam ab6588), fibrillin (Cosmobio LSL-LB-2297), and laminin beta 2 (Novus Biologicals NBP1-00904). After 1 h incubation with a secondary antibody, positive signaling was detected by chemiluminescent using an ECL substrate (GE Healthcare). Immunoblots were densitometrically analyzed using GE Image Quant LAS4000 luminescent image analyzer (GE Healthcare). The signal intensity of each sample was normalized to the total protein in its respective lane. Data are reported as mean ± SEM. The Kruskall-Wallis non-parametric test was used to assess differences among groups, and the Dunn's multiple comparison post-test was used when differences were observed. A *P* < 0.05 was considered significant.

## 3. Results and Discussion

**Figure 1:**
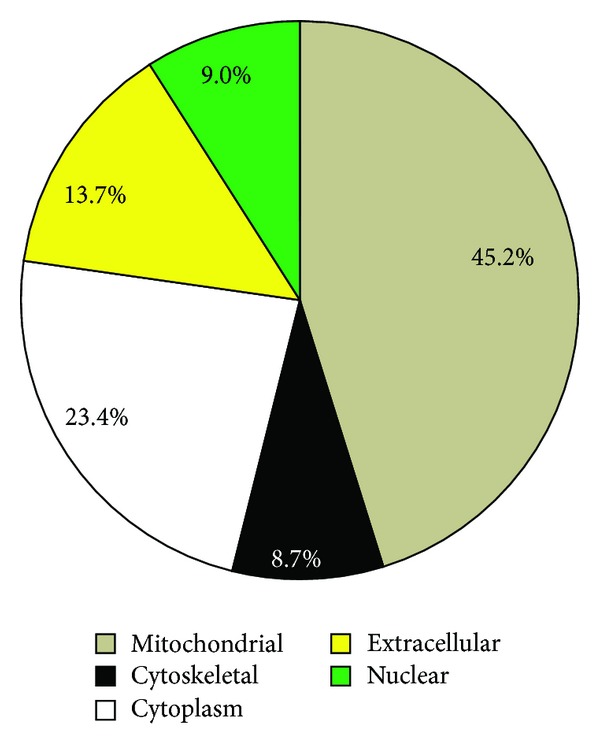
Decellularization of the left ventricles enriched for extracellular proteins, including secreted and membrane proteins. Using mass spectrometry, we identified a total of 321 proteins, of which 44 (13.7%) were extracellular proteins.

We identified 321 proteins by mass spectrometry, of which 44 proteins (13.7%) were extracellular, including secreted and cell membrane proteins (Figure 1). We used normalized spectrum counts to perform relative quantification and identify age- and SPARC-related changes (Table 1). In accordance with previous reports, the fibrillar collagens alpha 1(I) and alpha 2(I) showed an age-dependent increase that was blunted with SPARC deletion [9, 18, 24]. With age, the levels of total collagen and insoluble collagen, collagen fibril diameter, and the extent of collagen cross-linking all increase [9, 18, 24]. These changes lead to increased LV stiffness and cardiac dysfunction [25, 26]. Upon secretion, collagen molecules are processed and stabilized by the formation of covalent cross-links resulting in mature cross-linked collagen, processes influenced by SPARC [27]. The relationship between SPARC and postsynthetic procollagen processing suggests that SPARC deletion may diminish the decline in diastolic function observed with aging.

**Figure 2:**
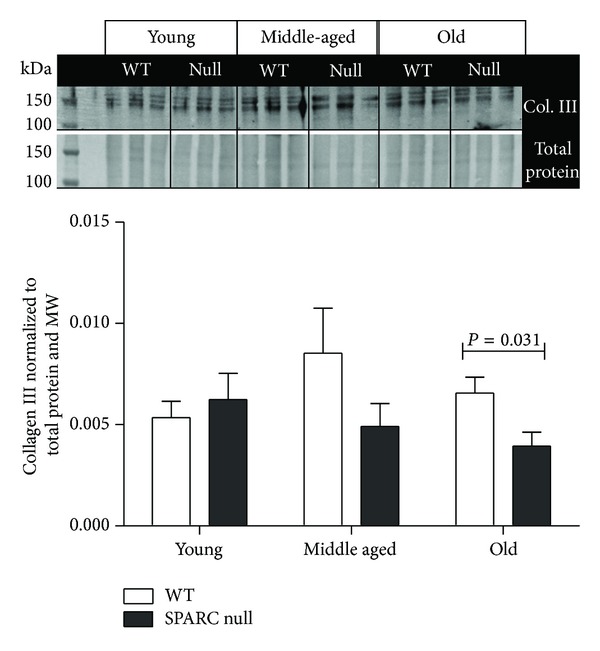
In the old mice, LV collagen III levels were lower in the SPARC null mice compared to the WT. Protein levels were quantified by immunoblot (*n* = 6/genotype/age). Two controls were used, total protein stain (loading control) and molecular weight marker (MW, blot to blot control). The signal intensity of the MW was used to normalize the data among blots, while protein levels were normalized to the total protein in its respective lane. Values were plotted as mean ± SEM.

We found that collagen III levels were reduced in the old null mice compared to WT (Figure 2). In a normal young adult heart, collagen III constitutes approximately 11% of the total cardiac collagen content [13]. Several studies have shown that during the aging process cardiac collagen III content gradually increases but at a lower rate when compared to collagen I [28, 29]. SPARC deletion or inhibition in the elderly may be a target of interest for the treatment of age-related cardiac fibrosis.

**Figure 3:**
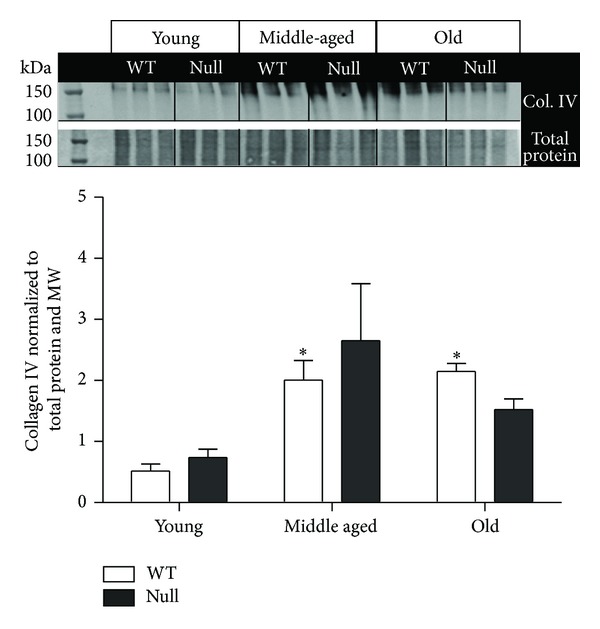
Collagen IV expression levels increased with age in the wild type (WT), but not in the SPARC-null (null) mice. Collagen IV protein levels were quantified by immunoblot (*n* = 6/genotype/age). Two controls were used, total protein stain (loading control) and molecular weight marker (MW, blot to blot control). The signal intensity of the MW was used to normalize the data among blots, while protein levels were normalized to the total protein in its respective lane. Values were plotted as mean ± SEM; *P < 0.05 versus respective young group.

Interestingly, the nonfibrillar collagen type IV increased with age in the WT mice but not in the SPARC null group (Figure 3). Collagen type IV is a reticular basement membrane type collagen that plays a fundamental role during embryonic cellular differentiation, proliferation, survival, and migration [30]. Collagen IV can form complex structural scaffolds, which are covalently linked and are required for basement membrane assembly [31, 32]. To date, there are no studies on collagen type IV in the aged heart; however, a study by Tarasov and colleagues identified a single-nucleotide polymorphism in the gene Col4a1 that was associated with increased central arterial stiffness in humans [33], suggesting that this protein plays an important role in cardiovascular function. The basal lamina surrounding cardiac myocytes contains collagen IV and these studies suggest that the myocyte basal lamina might thicken with age. Interestingly, laminin, nidogen, and perlecan—other significant components of basal lamina—did not exhibit differences in older versus younger tissues. Collagen type IV has also been reported to have a key role in the regulation of angiogenesis as assembly of the basal lamina by endothelial cells is a critical event in new blood vessel formation [34].* In vitro* studies have shown that collagen IV induces the formation of neovessels, stabilizes neovascular outgrowth, and prevents vascular regression [34]. The increase in collagen type IV, therefore, suggests an increased stimulus for angiogenesis with aging. Whether this results in an actual increase in vessel numbers needs to be evaluated.

**Figure 4:**
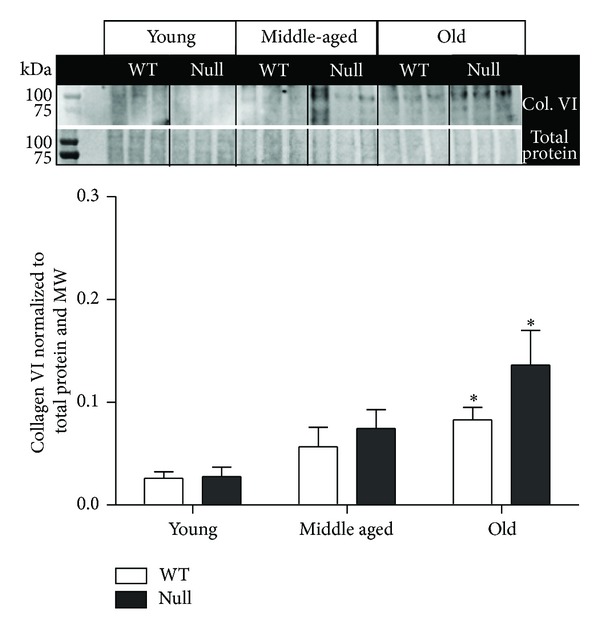
LV levels of collagen VI gradually increased with age. This increase was not SPARC dependent. Values were plotted as mean ± SEM; *n* = 6/genotype/age; *P < 0.05 versus respective young group.

Collagen type VI expression was also enhanced with age but differences in expression were not dependent upon SPARC expression (Figure 4). Type VI collagen molecules assemble end to end in a beaded filament arrangement [35, 36]. Typically this collagen is found in close proximity to collagens types I and III, forming a microfilament network with the fibrillar collagens [37]. Additionally, the N-terminus domain of collagen *α*1(VI) interacts with the C-terminus domain of collagen IV and in skeletal muscle colocalizes with collagen type IV [37, 38]. These data provide evidence that one of the key roles of collagen VI is to anchor the basement membrane to the underlying connective tissue. Aged-increased deposition of collagen VI may relate to decreased compliance of the left ventricular connective tissue.

Of note, the glycoprotein fibrillin-1 was increased in the middle-aged SPARC null mice. Fibrillin-1 is thought to act as a template for deposition of tropoelastin during elastic fibrogenesis [39]. Moreover, fibrillin-1 exists along individual microfibrils, facilitating their alignment into bundles and interaction with other ECM molecules [39, 40]. Even though fibrillin-1 levels returned to baseline (young levels) in the old null animals, this increase in protein during middle age is suggestive of increased microfibril deposition, which may benefit LV function as the heart ages.

One limitation of this study is that the extraction protocol prevented visualization of collagen degradation products that may occur with age. The decellularization process during the ECM-enrichment protocol removed soluble peptides present in the tissue. Additional studies focusing on the ECM degradome and the effects that these cleavage products may have on cardiac dysfunction with age are warranted.

## 4. Conclusions

In summary, our data suggest SPARC as a possible therapeutic target for aging induced cardiac dysfunction. Increases in fibrillar collagen with age have been previously reported. We also found age-dependent increases in two other types of collagen expressed in the heart, collagen IV and VI. Although the contribution of these nonfibrillar collagens to diastolic function is currently unknown, increases in collagen IV and VI might influence myocyte interaction with the interstitium. For example, increases in collagen IV might thicken the basal lamina of myocytes impeding extracellular communication between myocytes and fibroblasts and/or myocytes and surrounding vasculature. Collagen type VI has been proposed to influence ECM organization in and around bundles of muscle and vasculature. Hence increases in collagen VI might also affect cell to cell communication or cell to ECM interaction.

## Figures and Tables

**Table 1:** Quantitative values (normalized weighted spectra) for the identified extracellular proteins (*n* = 4  per group, values are mean ± SEM).

Identified Proteins	Molecular weight	Young WT	Young KO	Middle WT	Middle KO	Old WT	Old KO	Accession number
Secreted proteins								
Annexin A2	39 kDa	0	0	0	0	0.5 ± 0.5	0.5 ± 0.5	gi∣12849385
Apolipoprotein O	21 kDa	0	0.8 ± 1.0	0	0	0	0	gi∣123122452
Collagen type I alpha-1	138 kDa	10.0 ± 0.4	7.8 ± 2.6	12.5 ± 2.6	7.0 ± 0.8	10.5 ± 1.2	6.0 ± 1.8	gi∣34328108
Collagen type I alpha-2	130 kDa	6.0 ± 0.7	3.5 ± 1.3	9.5 ± 1.6	3.5 ± 1.3	9.3 ± 1.4	7.0 ± 0.6	gi∣111120329
Collagen type III alpha-1	139 kDa	0.3 ± 0	0.3 ± 0	1.5 ± 0.8	0.5 ± 0	1.3 ± 0	0.8 ± 0	gi∣74184771
Collagen type IV alpha-2	167 kDa	1.5 ± 0.6	0.8 ± 0.6	3.8 ± 0.6	2.3 ± 0.6	4.3 ± 0.8	3.0 ± 0	gi∣556299
Collagen type VI alpha-1	108 kDa	7.8 ± 0.6	8.8 ± 0.5	7.5 ± 1.2	7.8 ± 1.9	6.0 ± 1.1	6.3 ± 0.6	gi∣6753484
Collagen type VI alpha-2	110 kDa	4.0 ± 0.9	6.0 ± 1.2	7.8 ± 1.5	9.5 ± 1.4	11.0 ± 0.7	9.5 ± 1.7	gi∣22203747
Collagen type VI alpha-3	287 kDa	41.8 ± 6	46.3 ± 7	45.0 ± 7	51.5 ± 7	51.3 ± 5	48.0 ± 4	gi∣148708135
Collagen type XV	140 kDa	0	0	0	0.3 ± 0.3	0	0	gi∣11037306
Collagen type XXIV alpha-1	176 kDa	0	0	0.3 ± 0.3	0	0	0	gi∣116326001
Fibrillin-1	312 kDa	0	0.3 ± 0.3	0	0	0	0.5 ± 0.5	gi∣118197277
Fibrinogen, alpha	87 kDa	0.3 ± 0	0	0	1.0 ± 0.6	3.3 ± 1.7	3.0 ± 1.3	gi∣148683476
Fibrinogen, beta	55 kDa	0.0	0	0.3 ± 0	0.5 ± 0.5	1.3 ± 0.9	0.5 ± 0.5	gi∣33859809
Fibrinogen, gamma	49 kDa	0.0	0	0	0	0.5 ± 0.5	0	gi∣148683478
Laminin alpha-2	344 kDa	0.8 ± 0	1.5 ± 0.7	0.5 ± 0.5	0.3 ± 0.3	0	0.3 ± 0.3	gi∣117647249
Laminin beta-1	204 kDa	1.0 ± 0.7	0.5 ± 0.3	0.3 ± 0.3	0.3 ± 0.3	1.0 ± 0.6	0.3 ± 0.3	gi∣148704971
Laminin beta-2	197 kDa	0.3 ± 0.3	1.5 ± 0.9	0.5 ± 0.5	1.5 ± 0.6	0.5 ± 0.3	1.3 ± 0.5	gi∣31982223
Laminin gamma-1	179 kDa	1.8 ± 0.5	1.0 ± 0.4	2.0 ± 0.8	5.5 ± 1.2	2.3 ± 0.9	2.5 ± 0.6	gi∣148707495
Nidogen-1	137 kDa	1.0 ± 0.6	1.3 ± 0.5	0	0.3 ± 0.3	0.5 ± 0.5	0.8 ± 0.3	gi∣171543883
Perlecan	470 kDa	4.8 ± 0.5	4.8 ± 0.3	3.5 ± 1.0	5.5 ± 1.2	5.8 ± 0.8	6.8 ± 1.3	gi∣183979966
Prelamin-A/C	74 kDa	0.3 ± 0	0.8 ± 0.5	0.3 ± 0	0	0	0	gi∣162287370
Serum albumin	69 kDa	0	0	0	1.0 ± 0.5	0.3 ± 0.3	0	gi∣163310765
Troponin I, cardiac muscle	24 kDa	15.8 ± 0.9	13.5 ± 1.2	11.8 ± 2.1	14.3 ± 0.9	10.8 ± 2.6	9.8 ± 2.0	gi∣6678393
Troponin T2, cardiac	32 kDa	0.8 ± 0.5	1.0 ± 0.7	0.5 ± 0.5	0	0	0	gi∣148707615
Von Willebrand factor A domain-containing protein 8	213 kDa	2.3 ± 0.6	3.5 ± 0.6	1.8 ± 0.4	2.0 ± 0.5	3.0 ± 0.5	3.0 ± 0.8	gi∣226958579

Cell membrane proteins								
Alpha-sarcoglycan	43 kDa	0	0	0	0	0	0.3 ± 0.3	gi∣2411510
Aminopeptidase	103 kDa	0	0.5 ± 0.5	0.5 ± 0.5	1.5 ± 1.0	0	0.5 ± 0.5	gi∣1184161
ATP1a1 protein	108 kDa	5.3 ± 1.9	6.8 ± 2.9	4.3 ± 3.0	6.5 ± 3.8	2.0 ± 0.8	1.3 ± 0.8	gi∣16307541
ATP-binding cassette (ALD) 3	75 kDa	0	0	0	0	0	0	gi∣14318642
ATP-binding cassette (MDR/TAP) 8	78 kDa	0.8 ± 0.5	0.0	0.5 ± 0.5	0	0	0	gi∣148671187
EH domain-containing protein 4	61 kDa	2.0 ± 0.7	1.5 ± 0.9	2.8 ± 0.9	4.0 ± 0.4	1.5 ± 0.5	2.8 ± 0.3	gi∣31981592
Guanine nucleotide-binding protein, subunit beta-2	37 kDa	0	1.3 ± 0.6	0.5 ± 0.2	0.5 ± 0.3	0.5 ± 0.3	0	gi∣13937391
Guanosine diphosphate dissociation inhibitor 2	53 kDa	0	0	0	1.0 ± 1	0	0	gi∣148700276
Neutral cholesterol ester hydrolase 1	46 kDa	4.5 ± 0.3	3.0 ± 0.4	0	0.3 ± 0.3	0	0.5 ± 0.5	gi∣30520239
PDZ and LIM domain protein 5	24 kDa	2.5 ± 0.5	3.3 ± 0.6	1.5 ± 0.6	2.0 ± 0.4	0.5 ± 0.3	0.8 ± 0.8	gi∣300069034
Perilipin-4	139 kDa	3.5 ± 1.2	5.0 ± 0.8	4.8 ± 0.6	6.0 ± 2.0	8.0 ± 2.0	6.8 ± 1.6	gi∣157041252
Peroxisomal membrane protein 20	17 kDa	2.3 ± 0.3	3.5 ± 0.5	1.3 ± 1.3	1.5 ± 0.3	1.5 ± 0.5	1.5 ± 0.9	gi∣6746357
Platelet glycoprotein 4	53 kDa	0	1.3 ± 1.3	0.5 ± 0.5	0	0	0	gi∣74151899
Protease, serine 15	109 kDa	0.5 ± 0.5	0	0	0	0	0	gi∣148706233
Sodium/calcium exchanger 1	108 kDa	0.5 ± 0.5	0.8 ± 0.8	0	0	0	0	gi∣119120890
Sorbin and SH3 domain-containing protein 1	83 kDa	3.5 ± 1.6	4.0 ± 0.9	6.3 ± 0.6	5.0 ± 0.9	6.5 ± 0.9	5.3 ± 1.3	gi∣78000154
Transglutaminase	77 kDa	0	0	0	0	0	0.3 ± 0.3	gi∣201941
Tripartite motif-containing protein 72	53 kDa	6.5 ± 1.3	8.0 ± 0.8	10.0 ± 0.7	10.3 ± 1.8	7.8 ± 1.3	11.3 ± 1.1	gi∣121247302
